# Barriers to the provision of high-quality palliative care for people with dementia in England: a qualitative study of professionals' experiences

**DOI:** 10.1111/hsc.12094

**Published:** 2013-12-27

**Authors:** Nathan Davies, Laura Maio, Krish Vedavanam, Jill Manthorpe, Myrra Vernooij-Dassen, Steve Iliffe

**Affiliations:** 1 Research Department of Primary Care and Population Health, University College LondonLondon, UK; 2 Central and North West NHS London Foundation TrustLondon, UK; 3 Social Care Workforce Research Unit, King's College LondonLondon, UK; 4 Scientific Institute for Quality of Healthcare, Radboud University Nijmegen Medical CentreNijmegen, The Netherlands

**Keywords:** dementia, palliative care, qualitative research, quality of healthcare, terminal care

## Abstract

Approaches to palliative care that were originally developed for people with cancer are now being adopted for people with dementia, as a response to many reports of poor-quality care for people with dementia at the end of life. This study explored perceived barriers to the delivery of high-quality palliative care for people with dementia using semi-structured interviews. Recordings were transcribed verbatim and analysed using thematic analysis with an inductive approach and a coding strategy. To improve the trustworthiness of the analysis, independent reading and coding of the transcripts were undertaken, followed by discussions among the four researchers to reach agreement and consensus of the themes. Two group interviews (*n* = 7 and *n* = 6), 16 individual interviews and five interviews of pairs of professionals were conducted in 2011/2012 with participants from backgrounds in palliative care, dementia services, palliative care research and policy making. Four themes were identified as barriers to providing high-quality palliative care for people with dementia: (i) ambivalence towards the systematisation of palliative care; (ii) disconnection between services; (iii) different assumptions about training needs; and (iv) negotiation of risk. Understanding these barriers to providing high-quality palliative care for people with dementia could help in the development of a dementia-specific palliative care pathway.

## What is known about this topic

- People with dementia often do not receive or have access to palliative care services.
- Dementia training is needed for palliative care professionals and palliative care training is needed for dementia professionals.
- Declining communication abilities among people with dementia limit their access to quality palliative care.
- Palliative care has been presented as a form of euthanasia by sections of the media in the United Kingdom.

## What this paper adds

- Professionals are unsure as to how to organise palliative care for people with dementia because they are ambivalent about the necessary systematisation of care.
- When professionals want training, they are not always referring to the acquisition of new knowledge and skills.
- Professionals are fearful of the risks involved in providing palliative care for people with dementia.

## Introduction

The number of people in need of palliative care is steadily growing as the world's population grows and people live longer. Within this ageing population, the prevalence of age-related conditions such as dementia will rise. Approximately 800,000 people in the United Kingdom have dementia (Lakey *et al*. 2012), the number predicted to increase to over one million by 2025 (Knapp & Prince 2007). However, palliative care approaches were developed for people with terminal cancer and do not necessarily work well when applied to people with non-cancer conditions (Sampson *et al*. 2011). Palliative care for the purposes of this study was defined using the World Health Organization's (WHO) definition

Palliative care is an approach that improves the quality of life of patients and their families facing the problems associated with life-threatening illness, through the prevention and relief of suffering by means of early identification and impeccable assessment and treatment of pain and other problems, physical, psychosocial and spiritual. (WHO 2002, p. 84)

At first sight, this lack of transferability is puzzling, for symptoms commonly experienced by people with dementia at the end of life are very similar to symptoms of other life-threatening illnesses. They include swallowing difficulties, pain, shortness of breath, skin breakdown, poor nutrition, disturbed sleep, infections, urinary incontinence and constipation (Mitchell *et al*. 2009). However, many people with dementia experience these symptoms for longer periods of time compared with those with advanced cancer (McCarthy *et al*. 1997) and are less able to communicate distress or the effects of their symptoms. This prolonged and unpredictable dying trajectory also impacts the place of death, with UK hospices having few dementia patients (Sampson 2010, Kane 2012). People with dementia are most likely to die in care homes and hospitals (Kay *et al*. 2000, Mitchell *et al*. 2005, Handley *et al*. 2014); these settings might present different sets of challenges to the provision of good palliative care compared with hospices.

English practice guidelines recognise that the symptoms experienced by people with dementia require a palliative care approach (NICE and SCIE 2006). However, subsequent policy guidance for the English health service, such as the National Dementia Strategy, includes only minimal advice in this area (Banerjee 2009).

People with dementia often lack access to specialist palliative care, with most not having access to hospice services (Sampson *et al*. 2006). Several studies have explored the barriers and challenges to the delivery of quality palliative care for people with dementia (Sachs *et al*. 2004, Birch & Draper 2008, Thuné-Boyle *et al*. 2010, Harrison-Dening *et al*. 2012). One barrier is that dementia is often not recognised as a ‘terminal’ illness requiring palliation (Sachs *et al*. 2004). In addition, the course of dementia is unpredictable, making it difficult to reach an accurate prognosis (Sachs *et al*. 2004, Birch & Draper 2008), so recognition of the need for palliation of symptoms does not necessarily help with management at the end of life.

People with dementia experience communication problems with professionals, particularly in the advanced stages of dementia, which often makes the receipt and provision of care difficult (Birch & Draper 2008). Awareness of this communication deficit necessitates efforts to share decision-making with families and carers, but families report being given little information about what is happening, and likely to happen, to their relative (Thuné-Boyle *et al*. 2010). Harrison-Dening *et al*. (2012) also identified the absence of advanced care planning as a barrier to good care. Many professionals and families were unaware of its value, and care staff were calling emergency services for fear of ‘censure’ from authorities rather than acting in the person's best interests, even when advance plans and decisions had been formulated. Their caution may not be surprising in the context of palliative care being criticised as a form of euthanasia by sections of the media (O'Dowd 2012), prompting much debate within medicine (Boyd & Murray 2012).

This paper draws from the English data of a larger European project, the Implementation of Quality Indicators in Palliative Care Study (IMPACT), which aims to improve the organisation of palliative care. IMPACT is developing and testing quality indicator packages as tools for improving palliative care across settings and systems (Davies *et al*. 2014, Iliffe *et al*. 2013) and is being carried out in England, Germany, Italy, Norway and the Netherlands. The research question was: What are professional perspectives on barriers to the delivery of high-quality palliative care for people with dementia?

## Method

### Design

We used semi-structured individual face-to-face interviews to elicit in-depth understanding of relevant topics (Britten 1995). Group interviews were carried out if potential interviewees expressed a preference for them. The interview guide for the semi-structured interviews (Box 1) was developed from reviews of the literature (Raymond *et al*. 2014a,b) in consensus-seeking discussions across the research team (Davies *et al*. 2014).

**Box 1 Semi-structured interview schedule**

If you could recommend anything in your country that works well for people with dementia who are dying, what would that be?

If you can think of any area of care for people with dementia who are dying that needs to be improved the most, what would it be?

If you can think of something you would not recommend to other countries in relation to palliative care for patients with dementia in your country, what would that be?

How well do you think professionals collaborate with one another in palliative care for patients with dementia?

### Participants

Participants were identified using purposive sampling supported by snow-balling methods (Murphy *et al*. 1998) through dementia care organisations and from palliative care providers known to the multidisciplinary research team, using a sampling framework. The sampling framework consisted of a matrix of micro-, meso- and macro-level organisations working across primary, secondary and tertiary care settings, to capture different kinds of experiences and perspectives. The micro-level participants are clinical practitioners who provide dedicated palliative care within the settings. Meso-level participants are other services available not dedicated to palliative care, including service management. Finally, macro-level participants are those developing and implementing high-level guidelines and policies designed to support high-quality palliative care. Participants included national experts in policy, service managers and practitioners, patient and carer representatives, and researchers in palliative care. Individuals were invited to participate or to nominate someone whom they felt would be more appropriate.

### Procedure

The research was approved by (University College London) ethics committee. Participants were given a choice to be interviewed individually, in pairs or as a group, if more convenient. Verbal informed consent was received from all participants. Interviews took place in 2011/2012 at the professional's place of work or preferred location and varied in length from 20 to 60 minutes. Interviews were recorded with permission and field notes were made by the interviewers, or captured using contemporaneous notes when recording was not possible. Face-to-face interviews were preferred; however, two telephone interviews were conducted at participants' requests. Some asked to be interviewed with work colleagues, and these interviews were carried out in pairs or as a group. The interview schedule was adapted after the pilot interviews following discussions among the researchers. Assurances of confidentiality were provided to participants and all identifiers have been anonymised. Interviews were completed by an academic General Practitioner (GP) (SI) and a researcher with a psychology background (ND).

### Data analysis

Recordings were transcribed verbatim and analysed by four researchers (ND, SI, LM, KV) using thematic analysis, with an inductive approach and a coding strategy (Aronson 1994). To improve the trustworthiness of the analysis, independent reading and coding of the transcripts were undertaken, themes were regularly discussed among the four researchers to enhance the credibility of the results and rival explanations among the four researchers were explored until consensus was reached (Guba & Lincoln 1981, Mays & Pope 1995). The four researchers were from a range of backgrounds including anthropology, general practice, psychiatry and psychology, allowing for a range of perspectives when interpreting and discussing the data. Rival explanations and deviant cases were searched for within the data and discussed among the researchers to enhance the rigour of the results (Mays & Pope 2000).

## Results

Twenty-one interviews were conducted, with five interviews including two participants. We interviewed 18 clinical practitioners (five GPs, three old age psychiatrists, two palliative medicine consultants, one dementia nurse, four palliative care nurses and three research nurses), two researchers and six senior managers (one charity director, two policy advisors, one commissioning manager, one senior healthcare manager and one director of adult social services). As the participants fit into the sample frame, we interviewed 18 participants from the micro level, six participants from the meso level and eight participants from the macro level.

Two group interviews (*n* = 6 and *n* = 7) were convened from among staff working for a major care home company in England. The first group included two care home organisation directors and five senior care home managers, and the second group included four care home managers, one senior care home manager and one care home organisation director. This provided three participants from the macro level and ten participants from the meso level of the sampling frame. Recruitment continued until no new themes emerged from the data.

Four main themes emerged describing barriers to high-quality palliative care for people with dementia

- Ambivalence towards the systematisation of palliative care
- Disconnection between services
- Different assumptions about training needs
- Negotiation of risk.

### Ambivalence towards the systematisation of palliative care

The growing systematisation of palliative care for people with dementia dominated discussions. Systematisation in this context referred to the growing number of guidelines, standards, rules and regulations placed upon professionals in health and social care, making palliative care standardised leaving no room for flexibility. Views spanned a spectrum from a wish for rules (such as clinical guidelines) and boundaries (e.g. role demarcation within care pathways) that were thought to create a stable organisational structure and environment, to desires to be able to create a highly individualised care approach to capture an individual's needs, even if those needs sometimes could not be met by adhering to the rules. At this end of the spectrum, when interviewees referred to ‘boundaries’, which interfered with an individualised care pathway, they described limitations imposed by Health and Safety regulations, which actively interfered with delivery of good care.

Some nurses from community and hospital settings described feeling that palliative care for people with advanced cancer had become increasingly systematised, but inflexible, and expressed concern that this would occur for other conditions, including dementia. As a discipline, palliative care had started by ‘breaking rules’ and doing what was necessary to benefit the patient as shown by the quote below. Currently, practitioners felt that they had to follow guidelines and use prescribed tools and practice is audited and judged on performance

What I have seen over a 30-year period is a shift from that charismatic leadership to routinisation where it's just the same as every other service […]. It was a phenomenal change of approach when it first started [palliative care], it was about breaking the rules, breaking the boundaries, working at the edge all the time, […] there is nothing different, nothing is special about it anymore, so nobody is prepared to break the rules or bend the rules and everybody, because of the shift in clinical governance, the working guidelines, everybody is relatively obsessed with working within certain parameters […]. (Hospital-based Palliative Care Nurse 1)

Say, ‘Okay we have to think outside the box’, and I think that is a huge thing in end-of-life care. You can have your, ‘This is how it should be’, but when someone's dying, you, you have to be willing to give the extra or do something maybe slightly different […]. (Community-based Palliative Care Nurse 2)

[A patient] might have spent the last 20 years living on their sofa, but they're not allowed to die on their sofa. Or if they do, they're not allowed carers because they can't bend down to the sofa. And I don't know, it can be very frustrating sometimes. (Community-based Palliative Care Nurse 2)

However, those who were working at a step removed from ‘the frontline’ patient or resident care spoke of other rules and systems. These included the problematic divide in England between funding of services to meet healthcare needs under the National Health Service (NHS) and the means-tested localised system of social care. While NHS services are offered to people in care homes, many people in England pay for their place in a care home (self-funding). NHS middle managers felt that they had little influence on this care

We would have no jurisdiction over people who are self-funding and we [NHS Primary Care Trust] don't have a duty of care. (Commissioning Manager)

Many participants portrayed palliative care for people with dementia as chaotic and disorganised, with patients or residents not being seen by palliative care specialists, but rather by generalists, who sometimes struggled to know what to do for the best. Contrasts were drawn between patients with cancer receiving systematised palliative care, while those with dementia receive largely un-systematised care, with fewer resources available to them and many different professionals potentially involved, for example, mental health nurses, community nurses and GPs

Basically, if you've got a cancer then you're termed as palliative and everybody knows the input that you're going to get. But if you're coming to the end-of-life phase with complex conditions, then you don't come under the palliative care labels and you don't get the same level of care. (Senior Care Home Manager 1)

The interviews indicate that palliative care for people with dementia does not need to be systematised completely, but that an element of systematisation is wanted and needed. Participants felt that there was a need to incorporate elements of systematisation, such as the Gold Standards Framework (GSF) and Liverpool Care Pathway (LCP) into practice

[…] Liverpool Care Pathway and once somebody flashes that up, whether it be a family member or a nurse or a community worker, then it should be flashed up somewhere and then it all automatically brings a meeting. (Senior Care Home Manager 3)

[…] the tools are so valuable, things like the GSF, like the LCP, when you teach somebody and they have it, and it's there. (Research Nurse 3)

Yes and I think that actually having tools, you know, that they're very powerful. And, you know, things like the pain assessment, an embedded pain assessment tool that people are familiar with, that facilitates conversation with the GP. (Research Nurse 3)

Some tension was evident between expressed wishes for a set of rules, so professionals feel safe in what they are doing, and the view that the rules needed to be ‘flexible’. This perennial tension of discretion versus rule certainty is played out in professional roles which in social work terminology are known as ‘street-level bureaucracy’ (Lipsky 1980).

### Disconnection between services

As suggested above, palliative care for people with dementia was perceived by participants as fragmented and disjointed. Palliative care, by definition, should be holistic, but this is not what the participants experienced. In the view of participants, many professionals, such as GPs, or specialist palliative care professionals, were simply not being included in a patient's care and treatment decisions, and each professional group seemed able to diagnose that the fault lay elsewhere in the system

[…] a number of these admissions [to Hospital Accident and Emergency Departments (A&E)], I think, the last 10–15 years have almost doubled because you guys [GPs] are no longer being integrated within that pathway and you're no longer seeing them at home before they leave. (Old Age Psychiatrist 2)

[…] if they [generalists] don't have the skilled team around them to guide them, I mean I can quite see the, you know, ‘Oh actually I don't know what to do with this, I will just…’ (Care Home Director 1)

[…] we have a lot of nursing homes […] they don't even make the diagnosis of dementia. So they have a lot of patients who have no diagnosis. So they don't even get on the radar for care. And then when things go badly and they deteriorate, they get shipped into a hospital and they have an unfortunate death in A&E or on a medical ward, geriatric ward […]. (Palliative Medicine Consultant 1)

Some palliative care specialists felt it was important that if a patient was referred to them for additional care that others, such as the GP could not provide, they did not also transfer and relinquish responsibility to them, but instead remained in contact and therefore connected to the care of that patient

[answering if it would be acceptable for patients with palliative needs to be referred to them as specialists] I think yes as long as they [GPs] are also seeing that person and that kind of thing as well. (Community Palliative Care Nurse)

Nonetheless, participants (whether social care, healthcare specialists or generalists) expressed the view that they all need to ‘come together’ to ensure that a person's and families' complex needs at the end of life could be met

It's about joining it all up, isn't it? (Care Home Director 1)

However, a Commissioning Manager highlighted that joining services together was difficult when funding was fragmented

[…] that integrated pathway I think it's a real block because the funding is apportioned out. (Commissioning Manager)

Not surprisingly, as demonstrated above, it appeared that connection of services meant different things to different people. It seemed to be defined or exemplified as the seamless ‘joining up’ of social and healthcare, the building of relationships between staff to ensure communication within and across organisations/services, and collaboration between specialist and generalist services.

### Different assumptions about training needs

Participants seemed to describe ‘training’ in two different ways. Some talked about a lack of skills and acquiring enough skills to perform more tasks in a standard fashion, thus reducing the need to call for specialist help

I would like to be prepared for setting up a syringe driver really quickly and have a system in place for doing that, which is something I've asked our local palliative care team if they can provide direct training on that, so that it can happen really quickly if the need arises, because I don't know, I think most of the time the need isn't there, but I wouldn't like to feel uncomfortable about being a bit clumsy and slow about setting it up. (GP 1)

[…] doctors and nurses didn't actually have the skill base and the response base and the structures to enable them to be good. (Old Age Psychiatrist 2)

Training was also conceived as a tool for acquiring the confidence to perform tasks that participants felt unsure about. This meaning of ‘training’ reflected a lack of confidence in using the knowledge and skills with training being sought as a validation of experience

[…] there's a lot, a huge amount of experience out there, they just need a little bit of confidence to get past the first hurdle and there will be a lot of good knowledge about, you know, just basic approaches around dementia care […] people will start, you know, thinking about what they're doing when they're prescribing, what checks, you know. (GP 1)

The notion of training as an enabler of practice was not expressed uniformly across participants. Some suggested that it was not enough to develop training around promoting confidence or acquiring new skills, suggesting that there are other latent problems hidden within the term ‘training’. A minority thought information was available, but was not being used and maintained or that ‘people’ are simply not interested in palliative care

I did a quick guide for adding people to the palliative care registers, and because the Gold Standards Framework guidance that's been floating around for a long time, was – no one was using it. (GP 1)

[…] approach to assessing a – there are tools available that we can use. Have I ever used any of them? Not that I can recall. (GP 2)

In some cases, the lack of palliative care skills was not seen as a gap to be filled by the generalist, rather the responsibility of a specialist service

Like you get a lot of district nurses which, and I know GPs that are very much sort of, ‘If I wanted to do palliative care, I'd be a palliative care specialist’. (Community-based Palliative Care Nurse 2)

### Negotiation of risk

Those working within palliative care services and in dementia care are confronted by ‘risk’. Throughout the interviews, it was apparent that the way risk was perceived, and the extent to which individuals and groups were able to negotiate it and deal with it, all played a role in the development and delivery of palliative care for people with dementia. The interviews illustrated a wide spectrum of reactions to risk and its management. At one end of the spectrum, there was a desirable state of ‘trust’; where risk was well managed, there was good rapport between professionals, and between professionals and families. However, at the other end of the spectrum, participants mostly spoke of a state of uncertainty and hazards operating at various levels, leading to a lack of trust, a ‘fear’ of litigation, of threats to speciality and of blame

[…] where we struggle most at the moment is in communications between the nursing staff, the relatives and the medical staff. And we have a lot of difficulty sometimes in getting GP support that they will document that we've agreed that decision, they seem to be very reluctant to write anything down about the decision. And a lot of the decisions, our guidance is that they must be made by the medical officer, […] So the whole thing becomes a grey area where we talk to the relatives, but the GP doesn't support us in any way. So clear end of life decisions or ways forward are, are not, they're not clear any more, they're just grey areas, because there's not a consensus of opinion that the medical staff are signed up to. (Care Home Director 2)

Yes and when I said, ‘Look, you know, perhaps we should discuss this first’, [prescribing] or something. [Specialist Palliative Care Nurse] Said, ‘Well in that case I won't prescribe for them’, and sort of took his ball away and well that isn't going to work. So that's why I'm slightly wary of having these very vertical special teams, because it disempowers everybody else and everybody else will say, ‘Oh they'll do that then’. (GP 3)

A sense of insecurity added to a fragmented, conflictual professional domain which was perceived negativity

Yeah threats to specialism, threats to generalism, you know um professional rivalries and jealousies, um it's all there it's all out there. Yeah patients and relatives get exposed to all of it in all of those organisations. (Hospital-based Palliative Care Nurse 1)

Even when professionals, services and teams trusted one another, participants reported that families may not trust professionals

But I think more and more these days relatives actually are more demanding and have higher expectations and see people dying as a failure. (Senior Care Home Manager 2)

However, you've got family dynamics or family coming and visiting. And I'm just thinking about one particular case where the patient was having noisy breathing but they [patient] weren't distressed by it. There was a bit of excretions, okay, and they were so comatose that, you know, there wasn't a problem. But it was a huge problem for the family and that's the reason the syringe driver was set up on that person. (GP 1)

In the absence of trust, practitioners may develop a sense of threat, which can disable them. Working in the area of palliative care can lead to situations and decisions, which may be judged controversial in themselves. Clinicians and other professionals alike constantly feared legal challenges (even if this had never happened) if they acted against the wishes of families

[…] sometimes some doctors are so frightened about litigation, they're very quick to send that person off to hospital, to get rid of the responsibility that they can decide on syringe drivers or whatever they can use in hospital, it's out of their hands, because they are just so frightened of making that decision. (Senior Care Home Manager 2)

Well who's, who's decision is it whether this person goes to hospital? How do I make the decision? If I don't, if I think it's, there's a degree of medical futility, and it's in the patient's best interests to, where do I stand legally with that as a clinician? Where do I stand legally with that as a family member? (GP 2)

And a lot of staff are very frightened about doing the wrong thing I think sometimes, they're quite frightened about families. (Clinical Nurse Specialist 1)

## Discussion

This study has identified several barriers which professionals think obstruct the delivery of high-quality palliative care for people with dementia, from organisational barriers such as the lack of connection between services and the risks of systematisation of services, to more personal challenges of different meanings of sufficiency of training and negotiation of risk and fear. These reflect previously identified barriers (Sachs *et al*. 2004, Birch & Draper 2008, Thuné-Boyle *et al*. 2010), confirming that these are not simple barriers to resolve. We agree with Harrison-Dening *et al*. (2012) that many barriers are underpinned by feelings of uncertainty, including uncertainty about disease trajectory. However, the current study suggests that further issues of uncertainty apply to many aspects such as systematisation and not simply the disease trajectory. Fear also appears to underpin many barriers, which may be exacerbated by recent media and public criticism (O'Dowd 2012) of the ethics of palliative care approaches.

### Ambivalence towards the systematisation of palliative care

This study reveals health and social care system challenges to be present in England, which appear to be quite different from those described about financial disincentives in the United States, and independent of the problems of prognostication in dementia (Sachs *et al*. 2004). Some professionals considered that palliative care is becoming increasingly systematised, with rules and strict boundaries, even as it widens its remit to all life-limiting conditions. Practitioners’ calls for greater structure and clearer rules to guide palliative care for people with dementia co-existed with feared loss of flexibility in clinical practice. The views of participants in this study suggest that there should be some caution when systematising palliative care for people with dementia and that all care providers need to be fully engaged with this systematisation process so as to retain as much flexibility as possible (Lawrence *et al*. 2011). The recent controversy about the LCP illustrates this caution (Chinthapalli 2013, Torjesen 2013).

### Disconnection between services

Palliative care should be based on a multidisciplinary approach, where a range of professionals work together (Pastrana *et al*. 2008). Participants suggested that this remains an aspiration as services remain fragmented, supporting the call from the National Dementia Strategy for the construction of a clear, integrated dementia care pathway (Banerjee 2009). Harrison-Dening *et al*. (2012) argue that a lack of co-ordination, such as that offered by a care pathway, has a profoundly negative effect on the co-ordination of care provided, particularly at times of crisis.

### Different assumptions about training needs

Previously, studies have argued that more education is needed for both professionals and the wider community to improve awareness of dementia, together with more training for professionals to improve the delivery of palliative care for dementia (Sachs *et al*. 2004). However, in the current study, the term ‘training’ had two meanings, the acquisition of skills and the development of confidence, which itself refers to the validation of experiential knowledge. There was also recognition that some professionals do not want to work with palliative care or around death, and may claim a lack of knowledge or skill and refer patients to other services, so relinquishing responsibility. Some believe that inadequate training may reinforce the tendency to give responsibility to others (Gott *et al*. 2012). It appears that ‘training’ for all professionals and in all sectors should address confidence and fear as well as skill development; this may be best achieved through workplace learning. Despite increasing attention to palliative care within undergraduate medical and nursing curriculum (Sullivan *et al*. 2003), currently, few educational interventions have been developed and evaluated (Raymond *et al*. 2014b).

### Negotiation of risk

The findings of this study support those of Harrison-Dening *et al*. (2012) that professionals feel an element of fear (about the intervention of regulatory authorities and legal challenge) when dealing with the difficult decisions and situations that are often encountered when providing palliative care for people with dementia. However, unlike the Harrison-Dening *et al*. study, the present study suggests that fear is not just limited to social care staff. The hazards associated with dementia and palliative care, such as difficulties with prognostication, variable disease trajectories, problems with feeding and the impact on communication of declining cognitive capacity, create a risky environment for professionals. Professionals’ desires to acquire new skills in palliative care may also be affected by their mindfulness of the risks of harm, blame and litigation that they believe to be associated with their work. In discussing the theme of training, we identified lack of confidence as one meaning of ‘training’; lack of confidence can also overlap with feelings of fear.

### Strengths and limitations of the study

The sample within the current study is rather small and therefore only tentative conclusions can be drawn. Although a sampling framework of people from a variety of professions and care settings was used and a broad range and diversity of opinions were sought in this study, however, not all job roles involved in palliative care for people with dementia were included, for example, social workers or care managers, inspectors and regulators, and care home assistants.

All qualitative analysis is a process of reduction and it is recognised that this can compromise the totality of the qualitative data (Burnard 1998) and therefore nuanced opinions may have been lost. Finally, the results from this study only apply to England; other healthcare systems may not have the same features.

### Implications for policy, practice and research

The findings reported in this paper may be helpful to those developing palliative care services for people with dementia. They confirm that a wide range of professionals are working with people with dementia in many different settings There is little hospice care for people with dementia in England (Hughes *et al*. 2005, Sampson *et al*. 2006), and the care home sector is large, but varies in capacity, engagement with health professionals and skill mix (Lievesley *et al*. 2011). The dementia care workforce in social care (including care homes and home care) is the least qualified part of the sector (Hussein & Manthorpe 2011) and experiences high levels of staff turnover. Policy aspirations about training need to recognise this. Recent media criticism of palliative care pathways (Torjesen 2013) highlights the fear and risks that were evident in the interviews, but our data suggest that these also reflect general discomfort with services and systems, and are not simply about uncertainty related to prognosis as suggested by the research literature (Sachs *et al*. 2004, Birch & Draper 2008). While there is a growing body of research identifying the challenges to providing good care for a person with dementia, there is also a need for research to identify the characteristics of the practitioners, care settings and wider support systems that facilitate this. There will be further work to explore these facilitators in the IMPACT study.

## Conclusions

This paper has identified four main barriers to providing good-quality palliative care for people with dementia, which should be considered by those developing care pathways for this group. There is ambivalence towards the systematisation of palliative care; disconnection between services; different assumptions about training needs; and negotiation of risk. We suggest that these issues may only conceal much deeper issues, which should be considered in the context and underlying social relations that have given rise to them.

## References

[b1] Aronson J (1994). A pragmatic view of thematic analysis: The qualitative report. http://www.nova.edu/ssss/QR/BackIssues/QR2-1/aronson.html.

[b2] Banerjee S (2009). Living Well with Dementia: A National Dementia Strategy.

[b3] Birch D, Draper J (2008). A critical literature review exploring the challenges of delivering effective palliative care to older people with dementia. Journal of Clinical Nursing.

[b4] Boyd K, Murray S (2012). Using end of life care pathways for the last hours or days of life. British Medical Journal.

[b5] Britten N (1995). Qualitative research: qualitative interviews in medical research. British Medical Journal.

[b6] Burnard P (1998). Qualitative data analysis: using a word processor to categorise qualitative data in social science research. Social Science and Health.

[b7] Chinthapalli K (2013). The birth and death of the Liverpool Care Pathway. British Medical Journal.

[b8] Davies N, Maio L, Van Riet Paap J (2014). Quality palliative care for cancer and dementia in five European countries: some common challenges. Ageing and Mental Health.

[b9] Gott M, Seymour J, Ingleton C, Gardiner C, Bellamy G (2012). ‘That's part of everybody's job’: the perspectives of health care staff in England and New Zealand on the meaning and remit of palliative care. Palliative Medicine.

[b10] Guba E, Lincoln Y (1981). Effective Evaluation: Improving the Usefulness of Evaluation Results Through Responsive and Naturalistic Approaches.

[b11] Handley M, Goodman C, Froggatt K (2014). Living and dying: responsibility for end-of-life care in care homes without on-site nursing provision – a prospective study. Health and Social Care in the Community.

[b12] Harrison-Dening K, Greenish W, Jones L, Mandal U, Sampson EL (2012). Barriers to providing end-of-life care for people with dementia: a whole-system qualitative study. BMJ Supportive and Palliative Care.

[b13] Hughes JC, Robinson L, Volicer L (2005). Specialist palliative care in dementia. British Medical Journal.

[b14] Hussein S, Manthorpe J (2011). The dementia social care workforce in England: secondary analysis of a national workforce dataset. Aging and Mental Health.

[b15] Iliffe S, Davies N, Vernooij-Dassen M (2013). Modelling the landscape of palliative care for people with dementia: a European mixed methods study. BMC Palliative Care.

[b16] Kane M (2012). My Life Until the End: Dying Well with Dementia.

[b17] Kay DWK, Forster DP, Newens AJ (2000). Long-term survival, place of death, and death certification in clinically diagnosed pre-senile dementia in northern England. The British Journal of Psychiatry.

[b18] Knapp M, Prince M (2007). Dementia UK: A Report to the Alzheimer's Society on the Prevalence and Economic Cost of Dementia in the UK.

[b19] Lakey L, Chandarina K, Quince C, Kane M, Saunders T (2012). Dementia 2012: A National Challenge.

[b20] Lawrence V, Samsi K, Murray J, Harari D, Banerjee S (2011). Dying well with dementia: qualitative examination of end-of-life care. British Journal of Psychiatry.

[b21] Lievesley N, Crosby G, Bowman C, Midwinter E (2011). The Changing Role of Care Homes.

[b22] Lipsky M (1980). Street-Level Bureaucracy, Dilemmas of the Individual in Public Services.

[b23] Mays N, Pope C (1995). Rigour and qualitative research. British Medical Journal.

[b24] Mays N, Pope C (2000). Assessing quality in qualitative research. British Medical Journal.

[b25] McCarthy M, Addington-Hall J, Altmann D (1997). The experience of dying with dementia: a retrospective study. International Journal of Geriatric Psychiatry.

[b26] Mitchell SL, Teno JM, Miller SC, Mor V (2005). A national study of the location of death for older persons with dementia. Journal of the American Geriatrics Society.

[b27] Mitchell SL, Teno JM, Kiely DK (2009). The clinical course of advanced dementia. New England Journal of Medicine.

[b28] Murphy E, Dingwall R, Greatbatch D, Parker S, Watson P (1998). Qualitative research methods in health technology assessment: a review of the literature. Health Technology Assessment.

[b29] NICE and SCIE (2006). Dementia: Supporting People with Dementia and Their Carers in Health and Social Care.

[b30] O'Dowd A (2012). Liverpool care pathway: doctors speak out. British Medical Journal.

[b31] Pastrana T, Junger S, Ostgathe C, Elsner F, Radbruch L (2008). A matter of definition – key elements identified in a discourse analysis of definitions of palliative care. Palliative Medicine.

[b32] Raymond M, Warner A, Davies N, Manthorpe J, Ahmedzhai S, Iliffe S (2014a). Palliative care services for people with dementia: a synthesis of the literature reporting the views and experiences of professionals and family carers. Dementia.

[b33] Raymond M, Warner A, Davies N, Baishnab E, Manthorpe J, Iliffe S (2014b). Evaluating educational initiatives to improve palliative care for people with dementia: a narrative review. Dementia.

[b34] Sachs GA, Shega JW, Cox-Hayley D (2004). Barriers to excellent end-of-life care for patients with dementia. Journal of General Internal Medicine.

[b35] Sampson EL (2010). Palliative care for people with dementia. British Medical Bulletin.

[b36] Sampson EL, Gould V, Lee D, Blanchard MR (2006). Differences in care received by patients with and without dementia who died during acute hospital admission: a retrospective case note study. Age and Ageing.

[b37] Sampson EL, Burns A, Richards M (2011). Improving end-of-life care for people with dementia. British Journal of Psychiatry.

[b38] Sullivan AM, Lakoma MD, Block SD (2003). The status of medical education in end-of-life care: a national report. Journal of General Internal Medicine.

[b39] Thuné-Boyle ICV, Sampson EL, Jones L, King M, Lee DR, Blanchard MR (2010). Challenges to improving end of life care of people with advanced dementia in the UK. Dementia.

[b40] Torjesen I (2013). Bad press over Liverpool care pathway has scared patients and doctors, say experts. British Medical Journal.

[b41] WHO (2002). National Cancer Control Programmes: Policies and Managerial Guidelines.

